# Impacts of additive, dominance, and inbreeding depression effects on genomic evaluation by combining two SNP chips in Canadian Yorkshire pigs bred in China

**DOI:** 10.1186/s12711-022-00760-4

**Published:** 2022-10-22

**Authors:** Quanshun Mei, Zulma G. Vitezica, Jielin Li, Shuhong Zhao, Andres Legarra, Tao Xiang

**Affiliations:** 1 Key Laboratory of Agricultural Animal Genetics, Breeding and Reproduction of Ministry of Education & Key Laboratory of Swine Genetics and Breeding of Ministry of Agriculture, Huazhong Agricultural University, Wuhan, 430070 China; 2 INRAE, INP, UMR 1388 GenPhySE, 31326 Castanet-Tolosan, France

## Abstract

**Background:**

At the beginning of genomic selection, some Chinese companies genotyped pigs with different single nucleotide polymorphism (SNP) arrays. The obtained genomic data are then combined and to do this, several imputation strategies have been developed. Usually, only additive genetic effects are considered in genetic evaluations. However, dominance effects that may be important for some traits can be fitted in a mixed linear model as either ‘classical’ or ‘genotypic’ dominance effects. Their influence on genomic evaluation has rarely been studied. Thus, the objectives of this study were to use a dataset from Canadian Yorkshire pigs to (1) compare different strategies to combine data from two SNP arrays (Affymetrix 55K and Illumina 42K) and identify the most appropriate strategy for genomic evaluation and (2) evaluate the impact of dominance effects (classical’ and ‘genotypic’) and inbreeding depression effects on genomic predictive abilities for average daily gain (ADG), backfat thickness (BF), loin muscle depth (LMD), days to 100 kg (AGE100), and the total number of piglets born (TNB) at first parity.

**Results:**

The reliabilities obtained with the additive genomic models showed that the strategy used to combine data from two SNP arrays had little impact on genomic evaluations. Models with classical or genotypic dominance effect showed similar predictive abilities for all traits. For ADG, BF, LMD, and AGE100, dominance effects accounted for a small proportion (2 to 11%) of the total genetic variance, whereas for TNB, dominance effects accounted for 11 to 20%. For all traits, the predictive abilities of the models increased significantly when genomic inbreeding depression effects were included in the model. However, the inclusion of dominance effects did not change the predictive ability for any trait except for TNB.

**Conclusions:**

Our study shows that it is feasible to combine data from different SNP arrays for genomic evaluation, and that all combination methods result in similar accuracies. Regardless of how dominance effects are fitted in the genomic model, there is no impact on genetic evaluation. Models including inbreeding depression effects outperform a model with only additive effects, even if the trait is not strongly affected by dominant genes.

**Supplementary Information:**

The online version contains supplementary material available at 10.1186/s12711-022-00760-4.

## Background

Genomic selection (GS) [1, 2] has been intensively used in routine genomic evaluations of pigs, especially in developed agricultural countries [3]. In the Chinese pig industry, GS is a newly introduced technology, and a small number of pig companies have started applying GS as a routine genetic evaluation approach. Due to the different types of single nucleotide polymorphism (SNP) arrays available on the fiercely competitive market and the limited knowledge of the performance of these SNP arrays, many pig companies tend to use different SNP arrays to genotype their pigs in the initial stage of implementing GS. Consequently, pigs within one population can be genotyped with different SNP arrays. This has also been reported in a study on dairy cattle [4]. SNP arrays usually contain a large number of unique SNPs that are not shared with other chips. Thus, the integration of genomic information from different SNP arrays and the application of such information in pig genomic evaluation pose a challenge to these pig companies. The imputation of genotypes from a low-density to a high-density SNP panel is routinely performed [5, 6], providing a strategy for combining data from different SNP arrays for genomic evaluation. However, an appropriate strategy for integrating genomic information from different SNP arrays of medium density (i.e., 50K to 60K) for pig genomic evaluation has not yet been reported and deserves to be further investigated.

Although previous studies have demonstrated that dominance effects are not negligible [8], they are usually ignored in genetic evaluations because of the high computation requirements, and the large-scale datasets with high proportions of full sibs [7]. With the increases in computation ability and the availability of SNPs, it has become feasible to estimate dominance effects accurately [8, 9]. In previous studies, dominance effects have been fitted as a ‘genotypic’ (*biological*) effect (*d*) in linear mixed models. For example, SNPs are coded as 0, 1, and 2 for genotypes *AA*, *Aa*, and *aa*, respectively, and the coding of dominance effects is equal to 0, 1, and 0 for genotypes *AA*, *Aa*, and *aa*, respectively [8, 9]. In contrast, in traditional genetic evaluations, dominance effects are included in linear mixed models as dominant deviations. For instance, SNP dominance effects are coded as $-2{p}^{2},2pq,$ and $-2{q}^{2}$ for genotypes *AA*, *Aa*, and *aa*, respectively [10]. Vitezica et al. [10] referred to this parameterization as ‘classical’ (*statistical*). In our study, we used the terms ‘genotypic dominance effect’ and ‘classical dominance effect’ to refer to the dominance effects coded in either a genotypic manner or a dominant deviation manner, respectively, to avoid potential confusion.

An increasing number of studies have investigated the influence of including dominance effects in prediction models on genomic evaluations of livestock [8, 11–18]. When compared with a prediction model based on additive effects only, the models that included both additive and dominance effects perform at least as well as the additive model in genomic prediction and genomic mating [8, 9, 11, 14, 19] but require more computational resources. Nevertheless, as Xiang et al. [18] pointed out, when dominance effects are explicitly considered in a genomic model, it is essential to also include inbreeding depression effects to correctly estimate dominance variance and predict breeding values. To our knowledge, only a few studies have included inbreeding depression effects in the estimation of genetic variances, and the contributions of inbreeding depression effects to the genetic variance have generally been ignored. In addition, differences in genomic prediction between models including genotypic dominance effects and models including classical dominance effects have rarely been studied and need further investigation.

Thus, the objectives of our study were: (1) to explore an appropriate strategy and procedure for integrating genomic information from different SNP arrays (Affymetrix 55K and Illumina 42K) for further genomic evaluation; (2), to evaluate the impact of dominance effects and inbreeding depression effects on the estimates of the genetic variance and the genomic prediction of four production traits and one reproduction trait in Canadian Yorkshire pigs raised in China; and (3) to compare the models including different dominance effects (genotypic and classical) in terms of the accuracy of the prediction of breeding values. The work was performed in two stages: first, we determined the optimal imputation strategy and chip, and then, after collecting more data, we addressed the models including dominance effects.

## Methods

### Data

All the data were obtained from a national pig nucleus herd in North China. The herd’s purebred Yorkshire pigs were originally imported from Canada in 2014, and since then, the Yorkshire population within this herd has been continuously selected based on selection indices for five traits: average daily gain (ADG) in the range of 30 to 100 kg, backfat thickness (BF) at 100 kg body weight, loin muscle depth (LMD) at 100 kg body weight, days to 100 kg (AGE100), and the total number of piglets born (TNB) at first parity; in this study, we used phenotypic records for these five traits collected from 2012 to 2019 (those before 2014 were provided by the original Canadian breeding company (*Genesus*)): 38,785, 38,667, 38,644, 38,785, and 10,504 records were available for ADG, BF, LMD, AGE100, and TNB, respectively. All the phenotypic records for the four production traits were obtained at the same time point, allowing a 10-kg deviation from the final bodyweight ($100\pm 10$ kg). The pedigree was traced back to 2012, and included 326,576 pigs. Since the beginning of 2018, a limited number of DNA samples was collected from the tested pigs. As of May 2019, 2334 pigs have been genotyped. Each genotyped animal can be traced back at least four generations. Among these genotyped pigs, 1208 were genotyped with an Affymetrix 55K commercial array, and 1106 with an Illumina 42K array. Call rates higher than 90% were obtained for all individuals included in this study. Further quality control of each SNP array was performed as follows: SNPs with a call rate lower than 90% and SNPs with a minor allele frequency (MAF) lower than 0.05 were filtered out, and SNPs that deviated strongly from the Hardy–Weinberg equilibrium (p < 10^–7^) were removed. After quality control, 31,654 and 35,710 SNPs were retained from the Affymetrix 55K and Illumina 42K arrays, respectively. To avoid possible confusion in the subsequent analyses, we refer to the remaining genotypic data as Affymetrix 31K SNPs and Illumina 35K SNPs. Among these remaining SNPs, 23,430 (23K common SNPs) were shared by the two SNP arrays. In other words, after quality control, for the 1208 pigs genotyped with the Affymetrix array there were 31K SNPs, i.e. 23,430 (23K) common SNPs and 7724 (8K) Affymetrix array-specific SNPs; and for the 1106 pigs genotyped with the Illumina array there were 35K SNPs, i.e. 23,430 (23K) common SNPs and 12,280 (12K) Illumina array-specific SNPs.

### Imputation scenarios

**Fig. 1:**
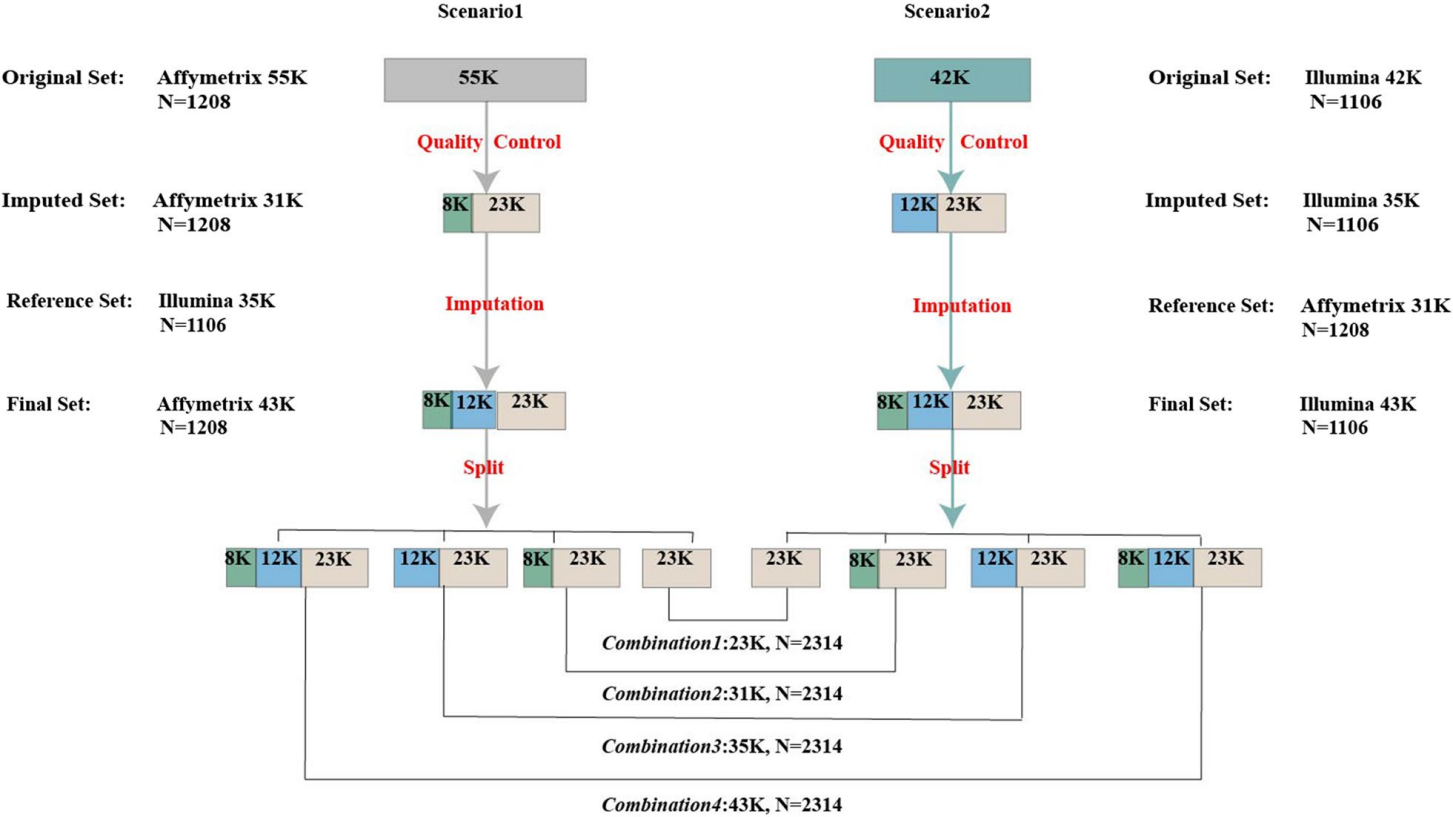
Two imputation scenarios and four combinations to integrate genomic information from the Illumina and Affymetrix arrays

To validate the imputation accuracy of the two SNP arrays, we tested two imputation scenarios. In Scenario 1, the 1106 pigs genotyped with the Illumina array (35K SNPs retained after quality control) were used as the reference set, and the remaining 1208 pigs genotyped with the Affymetrix array (31K SNPs retained after quality control) were used as the imputation set (Fig. 1). In contrast to Scenario 1, in Scenario 2 the 1208 pigs genotyped with the Affymetrix 31K SNPs were used as the reference set and the remaining 1106 pigs genotyped with the Illumina 35K SNPs were used as the imputed set. All the imputations were performed with the Beagle version 4.0 software [20], which can integrate genomic and pedigree information for imputation.

To use these genotypic data for genomic evaluation, four strategies to combine genomic information from the two chip arrays were investigated: *Combination 1*, included 2334 pigs and 23K SNPs, *Combination 2*, 2334 pigs and 31K SNPs, *Combination 3*, 2334 pigs and 35K SNPs and *Combination 4*, 2334 pigs and 43K SNPs. These four combinations are presented at the bottom of Fig. 1.

As mentioned above, 23K common SNPs were obtained in this study. To evaluate the imputation accuracy in Scenarios 1 and 2, 10% of the 23K common SNPs were masked (setting them to an ungenotyped state), and the masked SNPs were then imputed. After imputation, imputation accuracy was estimated by the concordance rate (CR) and the squared Pearson correlation coefficient (r^2^) between the genotyped and imputed SNPs. To reduce the systematic bias of evaluating imputation accuracy, we repeated the above procedure three times with different random seeds, leading to different subsets of the 10% masked SNPs. We obtained similar results across these repeats, and the average accuracies are presented in the “Results” section.

### Model-based reliability

To evaluate the effects of each strategy to combine the genomic information from two arrays on genomic evaluation, we applied the abovementioned four *combinations* to univariate single-step genomic best linear unbiased prediction (SSGBLUP) models for the five traits recorded in Canadian Yorkshire pigs [21, 22] and then evaluated the model-based reliabilities of each combined dataset.

For the four production traits, the model included sex, herd-year-season, and final body weight as fixed effects and an additive genetic effect, a common litter effect and a residual as random effects. For the reproduction trait, the model included herd-year-season as a fixed effect and an additive genetic effect and a residual effect as random effects. These analyses were carried out using the restricted maximum likelihood (REML) algorithm in the software DMU [23].

Individual model-based reliabilities were computed as follows [24]:

$${R}_{i}^{2}=1-\frac{{PEV}_{i}}{{\sigma }_{a}^{2}},$$

where ${R}_{i}^{2}$ is the reliability of individual $i$, ${\sigma }_{a}^{2}$ is the additive genetic variance, ${PEV}_{i}$ is the prediction error variance of the EBV of individual $i$, and is obtained by inverting the coefficient matrix of Henderson’s mixed model equations corresponding to the model used [24]. Note that this is an approximation because, strictly speaking, the denominator should be ${H}_{ii}{\sigma }_{a}^{2}$, where $\mathbf{H}$ is the combined pedigree-based and marker-based relationship matrix [21, 22]; in other words, genomic inbreeding is included [25]. Our approximation assumes ${H}_{ii}=1$ for all individuals to reduce the computational demand, which does not affect the comparison of the different strategies. The mean model reliability was calculated as the average ${\mathrm{R}}^{2}$ across all the pigs. Among the four strategies applied to combine the genomic information from the two SNP arrays, we defined that the optimal strategy was the combination that achieved the highest mean ${\mathrm{R}}^{2}$, and the subsequent analyses were based on this optimal combined dataset.

### Prediction models

Our results indicated that *Combination 3* was the optimal strategy for combining the information from the two arrays; thus, we advised the pig company to use the Illumina 42K SNP array for genotyping additional pigs so that we could estimate the dominance variance more accurately, and 4300 additional pigs with phenotypic records obtained between 2019 and 2020 were genotyped. These pigs were selected based on the following criteria: (1) they had progeny; (2) they were phenotyped for at least three of the target traits (ADG, BF, LMD, AGE100, and TNB); and (3) the total number of males or females within a litter was limited to four. Based on these criteria, we obtained 6614 genotyped pigs. The numbers of phenotypic records for ADG, BF, LMD, AGE100, and TNB were 41,367, 41,249, 41,224, 41,367, and 10,811, respectively (Table 1). In total, there were 467,244 pigs in the pedigree. Descriptive statistical data of the phenotypes are in Table 1. The mean pedigree-based inbreeding coefficient was 0.007 (ranging from 0 to 0.267).

**Table 1:** Descriptive statistics

Trait	Mean (SD)	Min.	Max.	Animals with phenotype
ADG (g)	623.3 (76.6)	298.6	1068.9	41,367
BF (mm)	13.9 (3.4)	5.0	32.0	41,249
LMD (mm)	58.7 (5.7)	30.2	90.0	41,224
AGE100 (days)	162.2 (11.9)	120.4	244.8	41,367
TNB	14.1 (3.5)	0.0	34.0	10,811

ADG: average daily gain; BF: backfat thickness; LMD: loin muscle depth; AGE100: days to 100 kg; TNB: total number of piglets born at first parity

These genotypic data were included in the single-step additive genetic evaluation model and were used to calculate the pre-corrected phenotypes of each trait. The pre-corrected phenotype (${\mathbf{y}}_{c}$) was calculated as ${\mathbf{y}}_{c}=\widehat{\mathbf{a}}+\widehat{\mathbf{e}}$, where $\widehat{\mathbf{a}}$ and $\widehat{\mathbf{e}}$ were the estimated additive genetic values and residuals for each tested pig. The pre-corrected phenotypes $({\mathbf{y}}_{c})$ of the 6614 genotyped pigs were used for the subsequent genomic prediction analysis. To evaluate the impact of dominance effects and inbreeding depression effects on genomic prediction, six genomic models were used to estimate variance components and predict total genetic effects as follows:

$$\begin{aligned}MA:&\quad {\mathbf{y}}_{{\varvec{c}}}=\bf{1}\mu +\mathbf{Z}\mathbf{a}+\mathbf{e},\\ MAD: &\quad{\mathbf{y}}_{{\varvec{c}}}=\bf{1}\mu +\mathbf{Z}\mathbf{a}+\mathbf{W}\mathbf{v}+\mathbf{e},\\ MA{D}^{*}:&\quad {\mathbf{y}}_{{\varvec{c}}}=\bf{1}\mu +\mathbf{Z}\mathbf{a}+\mathbf{W}{\mathbf{v}}^{\mathbf{*}}+\mathbf{e},\\ MAI: &\quad {{\varvec{y}}}_{{\varvec{c}}}=\bf{1}\mu +\mathbf{f}\eta +\mathbf{Z}\mathbf{a}+\mathbf{e},\\ MAID: &\quad {\mathbf{y}}_{{\varvec{c}}}=\bf{1}\mu +\mathbf{f}\eta +\mathbf{Z}\mathbf{a}+\mathbf{W}\mathbf{v}+\mathbf{e},\\ MAI{D}^{*}:&\quad {\mathbf{y}}_{{\varvec{c}}}=\bf{1}\mu +\mathbf{f}\eta +\mathbf{Z}\mathbf{a}+\mathbf{W}{\mathbf{v}}^{\mathbf{*}}+\mathbf{e},\end{aligned}$$

where ${\mathbf{y}}_{c}$ is the vector of pre-corrected phenotypes for each trait; $\mu$ is the overall mean; $\mathbf{f}$ is the vector of genomic inbreeding coefficients, calculated as $\bf{1}-\frac{{\varvec{h}}}{m}$, where **1** is a vector in which all elements are 1, $m$ is the number of SNPs, and $\mathbf{h}$ is a vector of the number of heterozygous loci for each individual [18]; $\eta$ is the inbreeding depression parameter; $\mathbf{a}$ is the vector of random additive genetic effects for each animal; $\mathbf{v}$ is the vector of random genotypic dominance effects for each individual; ${\mathbf{v}}^{\mathbf{*}}$ is the vector of random classical dominance deviation effects for each individual; $\mathbf{Z}$ and $\mathbf{W}$ are the corresponding incidence matrices; and **e** is the vector of residual effects. It was assumed that random effects followed normal distributions: $\mathbf{a}\sim N(\bf{0},\mathbf{G}{\sigma }_{a}^{2})$, $\mathbf{v}\sim N(\bf{0},\mathbf{D}{\sigma }_{d}^{2})$, ${\mathbf{v}}^{\mathbf{*}}\sim N(\bf{0},{\mathbf{D}}^{\mathbf{*}}{\sigma }_{{d}^{*}}^{2}),$ and $\mathbf{e}\sim N\left(\bf{0},\mathbf{I}{\sigma }_{e}^{2}\right)$, where $\mathbf{G}$ is the additive genomic relationship matrix, and ${\sigma }_{a}^{2}$ is the additive genetic variance; $\mathbf{D}$ is the genotypic dominance relationship matrix, and ${\sigma }_{d}^{2}$ is the genotypic dominance genetic variance; ${\mathbf{D}}^{\mathbf{*}}$ is the classical dominance relationship matrix, and ${\sigma }_{{d}^{*}}^{2}$ is the classical dominance genetic variance; $\mathbf{I}$ is the identity matrix, and ${\sigma }_{e}^{2}$ is the residual variance. Narrow-sense heritability was defined as the ratio between additive genetic variance and phenotypic variance (${h}_{a}^{2}=\frac{{\sigma }_{a}^{2}}{{\sigma }_{p}^{2}}$), and the proportions of genotypic dominance variation and classical dominance variation to phenotypic variance were defined as ${h}_{d}^{2}=\frac{{\sigma }_{d}^{2}}{{\sigma }_{p}^{2}}$ and ${h}_{d*}^{2}=\frac{{\sigma }_{d*}^{2}}{{\sigma }_{p}^{2}}$, respectively.

The genomic relationship matrices for additive effects ($\mathbf{G}$), genotypic dominance effects ($\mathbf{D}$), and classical dominance effects (${\mathbf{D}}^{\mathbf{*}}$) were constructed as follows [8, 10, 26]:

$$\mathbf{G}=\frac{\mathbf{M}{\mathbf{M}}^{\boldsymbol{^{\prime}}}}{\sum_{j=1}^{m}2{p}_{j}{q}_{j}},$$

$$\mathbf{D}=\frac{\mathbf{K}{\mathbf{K}}^{\boldsymbol{^{\prime}}}}{2\sum_{j=1}^{m}{p}_{j}{q}_{j}(1-2{p}_{j}{q}_{j})},$$

$${\mathbf{D}}^{\mathbf{*}}=\frac{{\mathbf{K}}^{\boldsymbol{*}}{\mathbf{K}}^{\boldsymbol{*}\boldsymbol{^{\prime}}}}{\sum_{j=1}^{m}{(2{p}_{j}{q}_{j})}^{2}},$$

where $m$ is the number of SNPs; ${p}_{j}$ is the frequency of the reference allele at marker $j$; ${q}_{j}$ = 1 − ${p}_{j}$; $\mathbf{M}$ is a matrix with dimensions equal to the number of individuals and the number of SNPs, and the entries in column $j$ are 2–2${p}_{j}$, 1–2${p}_{j}$, and 0–2${p}_{j}$, corresponding to the genotypes *AA*, *Aa*, and *aa*, respectively; $\mathbf{K}$ is a matrix with dimensions equal to the number of individuals and the number of SNPs, and the entries in column $j$ are 0–2${p}_{j}{q}_{j}$, 1–2${p}_{j}{q}_{j},$ and 0–2${p}_{j}{q}_{j}$, corresponding to the genotypes *AA*, *Aa*, and *aa*, respectively; and the entries of ${\mathbf{K}}^*$ in column $j$ are − 2${{q}_{j}}^{2}$, 2${p}_{j}{q}_{j}$, and −2${{p}_{j}}^{2}$, corresponding to the genotypes *AA*, *Aa*, and *aa*, respectively. Estimation of the variance components generated by REML and predictions were carried out with the software DMU [23].

In addition, the goodness-of-fit of the models was measured using − 2 times the maximum log likelihood of each model ($log(likelihood)$). For the comparisons of the nested models (e.g., MA vs. MAD, or MA vs. MAD*), the superiority of the more complex model over the less complex one was examined by the likelihood ratio test (LRT), which was calculated as LRT = − 2 log(likelihood for Model 1) − (− 2 log(likelihood for Model 2). These differences were assumed to follow a mixture of a ${\chi }^{2}$ distribution with 1 degree of freedom and a peak at 0 [27, 28]. To compare the non-nested models (e.g., MAD vs. MAD*), we used Akaike’s information criterion ($AIC$), calculated as $AIC=2k-2\mathrm{log}(likelihood)$ (the lowest $AIC$ is the better), where $k$ is the number of estimated parameters. Finally, to compare the two models that included or not genomic inbreeding (e.g., MA vs. MAI), we used the Wald test to test the significance of the covariate of the inbreeding depression effect. The Wald statistic is $\frac{{\widehat{\eta }}^{2}}{\mathrm{Var}(\widehat{\eta })}$, where $\widehat{\eta }$ is the regression coefficient of the inbreeding depression effect. The regression coefficients and their variance were obtained by solving mixed model equations with the DMU software [23]. The Wald statistic followed approximately a Chi-squared distribution with 1 degree of freedom.

As pointed out by Christensen et al. [29], the contributions of the inbreeding depression effects to the genetic variance are not negligible in the MAID model. To better understand the contributions of the inbreeding depression effects to the genetic variance, the genomic inbreeding coefficients were reformulated as ${\mathbf{f}}={\bf{1}}-\frac{{\mathbf{N1}}}{m}$, where $\mathbf{N}$ is a matrix with dimensions equal to the number of individuals multiplied by the number of SNPs; the entries in the $\mathbf{N}$ matrix are 0, 1, and 0 for the genotypes *AA*, *Aa*, and *aa*, respectively; **1** is a vector of 1 s of corresponding length; and $m$ is the number of SNPs. As shown by Xiang et al. [18], the sum of the inbreeding depression effects and dominance genetic effects is calculated as follows:

$$\mathbf{f}\eta +\mathbf{v}=\left(\widetilde{\bf{1}}-\frac{\mathbf{N}\bf{1}}{m}\right)\eta +\mathbf{N}\mathbf{d}=\widetilde{\bf{1}}\eta +\mathbf{N}\left(\left(-\frac{\eta }{m}\right)\bf{1}+\mathbf{d}\right),$$

where $\mathbf{d}$ is a vector of the centered genotypic dominance effects for each SNP. This equation results in the actual dominance genetic effects becoming $(-\frac{\eta }{m})\bf{1}+\mathbf{d}$, where the mean of the dominance genetic effects is $-\frac{\eta }{m}$. Hence, the vector of the allele substitution effects ($\alpha$) is ${\varvec{\upalpha}}=\mathbf{a}+((-\frac{\eta }{m})\bf{1}+\mathbf{d})\left(\mathbf{q}-\mathbf{p}\right)$, where $\mathbf{a}$ is the vector of additive genetic effects, $\mathbf{p}$ is the vector of the frequency of the reference allele, and $\mathbf{q}=\bf{1}-\mathbf{p}$.

Thus, the estimates of the additive genetic variance (${\sigma }_{A}^{2}$) in the MAID model can be calculated following Christensen et al. [29]:

$$\begin{aligned}{\sigma }_{A}^{2}&=\sum_{j=1}^{m}{2{p}_{j}{q}_{j}E[\left({\alpha }_{j}\right)}^{2}] \\ &=\sum_{j=1}^{m}{2{p}_{j}{q}_{j}E\big[\left({a}_{j}+({d}_{j}-\frac{\eta }{m})*\left({q}_{j}-{p}_{j}\right)\right)}^{2}\big] \\&=\sum_{j=1}^{m}2{p}_{j}{q}_{j}\left({{\sigma }_{a}^{2}}_{j}+{{\sigma }_{d}^{2}}_{j} {\left({q}_{j}-{p}_{j}\right)}^{2}\right)+\sum_{j=1}^{m}{2{p}_{j}{q}_{j}\left({q}_{j}-{p}_{j}\right)}^{2}\left(\frac{{\eta }^{2}}{{m}^{2}}\right) \\ &={\sigma }_{a}^{2}+\sum_{j=1}^{m}{2{p}_{j}{q}_{j}\left({q}_{j}-{p}_{j}\right)}^{2}\left(\frac{{\eta }^{2}}{{m}^{2}}\right),\end{aligned}$$

where ${\alpha }_{j}$ is the substitution effect at marker $j$; ${a}_{j}$ is the additive genetic effect at marker $j$*;* ${d}_{j}$ is the dominance genetic effect at marker $j$; ${{\sigma }_{a}^{2}}_{j}$ is the additive genetic variance at marker $j$; ${{\sigma }_{d}^{2}}_{j}$ is the dominance genetic variance at marker $j$; and ${\sigma }_{a}^{2}$ is the estimated additive genetic variance obtained via REML. Previous studies [14, 18] have neglected the contribution of $\frac{{\eta }^{2}}{{m}^{2}}$, which is equal to $\sum_{j=1}^{m}{2{p}_{j}{q}_{j}\left({q}_{j}-{p}_{j}\right)}^{2}(\frac{{\eta }^{2}}{{m}^{2}})$.

In terms of directional dominance effects, the mean directional dominance effects of the markers is $-\frac{\eta }{m}$, and the directional dominance effect at marker $j$ is ${d}_{j}-\frac{\eta }{m}$. Thus, the estimates of the genotypic dominance genetic variance (${\sigma }_{D}^{2}$) in the MAID model can be calculated as follows:

$$\begin{aligned}{\sigma }_{D}^{2}&=\sum_{j=1}^{m}{2{p}_{j}{q}_{j}E\big[\left({d}_{j}-\frac{\eta }{m}\right)-\left(-\frac{\eta }{m}\right)\big]}^{2}\\ &=\sum_{j=1}^{m}{{2{p}_{j}{q}_{j}\sigma }_{d_{j}}^{2}}={\sigma }_{d}^{2},\end{aligned}$$

where ${\sigma }_{d}^{2}$ is the estimated genotypic dominance genetic variance obtained via REML. Hence, the inbreeding depression effects do not contribute to the genotypic dominance genetic variance.

The derivation of the additive genetic variance in model MAI is similar to that in model MAID, except that ${{\sigma }_{d}^{2}}_{j}$ is set to zero in model MAI. Nevertheless, the inbreeding depression term $\sum_{j=1}^{m}{2{p}_{j}{q}_{j}\left({q}_{j}-{p}_{j}\right)}^{2}(\frac{{\eta }^{2}}{{m}^{2}})$ needs to be considered. To better show the influence of the inbreeding depression effects across different traits, the estimates of the inbreeding depression coefficients were divided by the phenotypic standard deviation of the trait [30].

To reveal the differences in predictive abilities between the genomic models, the data were divided into a training dataset and a validation dataset based on a cutoff date of January 1, 2020. The numbers of pigs in the training and validation datasets for each trait are in Table 2. Predictive abilities were calculated as the correlation between the predicted total genetic values ($\widehat{\mathbf{g}}$) and the corrected phenotypes (${\mathbf{y}}_{c}$) in the validation dataset. In model MA, $\widehat{\mathbf{g}}$ was equal to the additive genetic effects $(\widehat{\mathbf{g}}=\widehat{\mathbf{a}})$; in model MAD, $\widehat{\mathbf{g}}$ was calculated as the sum of the additive genetic effects and genotypic dominance effects $(\widehat{\mathbf{g}}=\widehat{\mathbf{a}}+\widehat{\mathbf{v}})$; in model MAD*, $\widehat{\mathbf{g}}$ was calculated as the sum of the additive genetic effects and classical dominance deviation effects $(\widehat{\mathbf{g}}=\widehat{\mathbf{a}}+\widehat{{\mathbf{v}}^{\mathbf{*}}})$; in model MAI, $\widehat{\mathbf{g}}$ was calculated as the sum of the additive genetic effects and inbreeding depression effects $(\widehat{\mathbf{g}}=\mathbf{f}\widehat{\eta }+\widehat{\mathbf{a}})$; in model MAID, $\widehat{\mathbf{g}}$ was calculated as the sum of the additive genetic effects, genotypic dominance effects, and inbreeding depression effects $(\widehat{\mathbf{g}}=\mathbf{f}\widehat{\eta }+\widehat{\mathbf{a}}+\widehat{\mathbf{v}})$; and in model MAID*, $\widehat{\mathbf{g}}$ was calculated as the sum of the additive genetic effects, classical dominance deviation effects, and inbreeding depression effects $(\widehat{\mathbf{g}}=\mathbf{f}\widehat{\eta }+\widehat{\mathbf{a}}+{\widehat{\mathbf{v}}}^{*})$. Furthermore, the unbiasedness of the genomic predictions in each model was assessed according to the regression coefficient of ${\mathbf{y}}_{c}$ on $\widehat{\mathbf{g}}$, with an expected result of 1.

**Table 2:** Number of genotyped animals for each studied trait in the training and validation datasets

Trait	Training size	Validation size
ADG	5245	1311
BF	5231	1308
LMD	5223	1306
AGE100	5246	1312
TNB	3874	969

ADG: average daily gain; BF: backfat thickness; LMD: loin muscle depth; AGE100: days to 100 kg; TNB: total number of piglets born at first parity

## Results

### Imputation accuracies

In this study, imputation accuracy was assessed based on two statistics: CR and ${r}^{2}$. For CR, the mean imputation accuracies in Scenario 1 and Scenario 2 were equal to 0.957 and 0.956, respectively. For ${r}^{2}$, the mean imputation accuracies in Scenario 1 and Scenario 2 were equal to 0.923 and 0.908, respectively. The standard errors were smaller than 0.001. Overall, the imputation accuracy in Scenario 1 was slightly higher than that in Scenario 2.

### Model-based reliability

For the four *combinations* analyzed, the model-based reliabilities (${\mathrm{R}}^{2}$) for the five studied traits are in Table 3. For each trait, ${\mathrm{R}}^{2}$ was similar for the four *combinations*. Since *Combination 3* was slightly more accurate than any other combination, *Combination 3* was selected for subsequent genomic evaluations in this study.

**Table 3:** Reliability of the different combinations to integrate genomic information from the Illumina and Affymetrix arrays based on the SSGBLUP model

Trait	Combination 1	Combination 2	Combination 3	Combination 4
ADG	0.400	0.400	0.401	0.400
BF	0.414	0.414	0.415	0.414
LMD	0.388	0.388	0.390	0.389
AGE100	0.367	0.366	0.368	0.367
TNB	0.173	0.174	0.176	0.174

ADG: average daily gain; BF: backfat thickness; LMD: loin muscle depth; AGE100: days to 100 kg; TNB: total number of piglets born at first parity

### Estimation of variance components

Estimates of narrow-sense heritabilities and the proportions of dominance variations relative to phenotypic variance are in Table 4, and the estimates of each variance component and the ratio of dominance to total genetic variance are in Additional file 1: Table S1. For each trait, the estimated additive genetic variances were similar across the six models regardless of whether nonadditive effects were included or not. The four production traits (ADG, BF, LMD, and AGE100) showed moderate narrow-sense heritabilities ranging from 0.210 to 0.373, and the reproduction trait TNB had a low narrow-sense heritability ranging from 0.093 to 0.102. TNB exhibited higher ratios of genotypic and classical dominance to total genetic variance (see Additional file 1: Table S1), ranging from 0.108 to 0.123 and from 0.182 to 0.203, respectively, than the production traits (ranging from 0.018 to 0.105). However, for all traits, there were no differences between the proportions of classical and genotypic dominance variation when standard errors were taken into account.

**Table 4:** Estimates of the heritabilities and inbreeding depression ($\eta$) and their standard error (SE) for each trait and each genomic model

Trait	Model	${\mathrm{h}}_{a}^{2}(\mathrm{SE})$	${{\mathrm{h}}_{d}^{2}(\mathrm{SE})|\mathrm{h}}_{{d}^{*}}^{2}(\mathrm{SE})$	$\eta (\mathrm{SE})$	$\eta /{\upsigma }_{p}$
ADG	MA	0.258 (0.018)			
	MAD	0.255 (0.018)	0.01 (0.011)		
	MAD*	0.258 (0.018)	0.008 (0.008)		
	MAI	0.262 (0.018)		− 229.58 (46.688)	− 4.023
	MAID	0.26 (0.018)	0.005 (0.011)	− 231.88 (48.422)	− 4.063
	MAID*	0.261 (0.018)	0.004 (0.008)	− 231.984 (48.59)	− 4.065
BF	MA	0.373 (0.019)			
	MAD	0.365 (0.019)	0.018 (0.011)		
	MAD*	0.37 (0.018)	0.014 (0.008)		
	MAI	0.374 (0.019)		− 4.749(2.195)	− 1.702
	MAID	0.366 (0.019)	0.017 (0.011)	− 4.899 (2.107)	− 1.756
	MAID*	0.371 (0.018)	0.013 (0.008)	− 4.992(2.046)	− 1.789
LMD	MA	0.249 (0.018)			
	MAD	0.243 (0.018)	0.012 (0.012)		
	MAD*	0.248 (0.018)	0.009 (0.009)		
	MAI	0.248 (0.018)		− 11.604 (3.78)	− 2.516
	MAID	0.243 (0.018)	0.011 (0.012)	− 11.696 (4.086)	− 2.536
	MAID*	0.246 (0.018)	0.008 (0.009)	− 11.696 (4.102)	− 2.536
AGE100	MA	0.218 (0.017)			
	MAD	0.21 (0.018)	0.025 (0.013)		
	MAD*	0.217 (0.017)	0.021 (0.01)		
	MAI	0.219 (0.017)		26.145 (6.628)	3.261
	MAID	0.213 (0.018)	0.02 (0.013)	28.143 (7.546)	3.51
	MAID*	0.218 (0.017)	0.017 (0.01)	28.307 (7.675)	3.53
TNB	MA	0.101 (0.016)			
	MAD	0.093 (0.016)	0.024 (0.018)		
	MAD*	0.101 (0.015)	0.014 (0.013)		
	MAI	0.102 (0.016)		− 7.278 (3.174)	− 2.197
	MAID	0.095 (0.017)	0.021 (0.018)	− 7.606 (3.51)	− 2.296
	MAID*	0.102 (0.016)	0.012 (0.013)	− 7.56 (3.444)	− 2.282

${\mathrm{h}}_{a}^{2}$: additive heritability; ${\mathrm{h}}_{d}^{2}=\frac{{\upsigma }_{d}^{2}}{{\upsigma }_{a}^{2}+{\upsigma }_{d}^{2}+{\upsigma }_{e}^{2}}$: genotypic dominance effect heritability; ${\mathrm{h}}_{{d}^{*}}^{2}=\frac{{\upsigma }_{{d}^{*}}^{2}}{{\upsigma }_{a}^{2}+{\upsigma }_{{d}^{*}}^{2}+{\upsigma }_{e}^{2}}$: classical dominance effect heritability; ${\sigma }_{p}$ is the square root of total phenotypic variance; MA: additive model; MAI: additive plus inbreeding depression model; MAD: additive plus genotypic dominance model; MAD*: additive plus classical dominance model; MAID: additive plus inbreeding depression plus genotypic dominance model; MAID*: additive plus inbreeding depression plus classical dominance model; ADG: average daily gain (g); BF: backfat thickness (mm); LMD: loin muscle depth (mm); AGE100: days to 100 kg; TNB: total number of piglets born at first parity

In models MAID and MAI, the contribution of the inbreeding depression effect to the additive genetic variance has been ignored in many previous studies e.g. [9, 18], and was found to be relatively small in our study. As shown in Additional file 1: Table S2, in the MAID model, the proportions of additive genetic variance contributed by the inbreeding depression effects relative to the total additive genetic variance were equal to 0.308%, 0.031%, 0.130%, 0.282%, and 0.273% for ADG, BF, LMD, AGE100, and TNB, respectively.

### Goodness-of-fit

The goodness-of-fit values of the six genomic models are in Additional file 1: Tables S1, S3–S5. The smaller the − 2 log likelihood value or the $AIC$ value is, the better is the model fit. For all traits except AGE100, the models without inbreeding depression effects (MA, MAD and MAD*) exhibited similar − 2 log likelihood values (see Additional file 1: Table S1), and the models with inbreeding depression effects (MAI, MAID and MAID*) also presented similar − 2 log likelihood values. Thus, the inclusion of dominance did not improve the goodness-of-fit of the models, except for AGE100 (MA vs. MAD, MAD*). Nevertheless, as shown in Additional file 1: Table S4, the significance of the dominance effects for this trait decreased when inbreeding depression was fitted in the model (MAI vs. MAID, MAID*), which implies that the effect of inbreeding depression (which is a significant effect for this trait) partly captured the dominance effects.

In addition, for the non-nested models (such as MAD vs. MAD*, MAID vs. MAID*), each paired group showed similar $AIC$ values for all traits, which indicated that different types of dominance effects (either ‘classical’ or ‘genotypic’) did not affect the goodness-of-fit of the genomic models.

### Estimation of the inbreeding depression parameter

The mean genomic-based inbreeding coefficient was 0.67 (ranging from 0.55 to 0.763). Estimates of the inbreeding depression parameter ($\eta$) in models MAI, MAID, and MAID* are in Table 4, and were all significantly different from 0. The Wald test showed significant differences at the 0.05 level of type 1 error of inbreeding depression effects (see Additional file 1: Table S5) between the models without inbreeding depression effects (MA, MAD and MAD*) and the corresponding models with inbreeding depression effects (MAI, MAID and MAID*), which showed that fitting inbreeding depression improved the goodness-of-fit of the models. In addition, there were no large differences in the estimates of the inbreeding depression parameter among models MAI, MAID and MAID*. For all traits except BF, the effects of inbreeding depression were detrimental, and they deviated significantly from zero at the 0.05 level of type 1 error based on the results of the Wald test. For instance, for ADG in the MAI model, $\eta$ was estimated to be − 229.9 g, which means that an increase of 0.10 in inbreeding led to a decrease of 22.9 g in the daily gain. In addition, in model MAI, the ratios of the inbreeding depression estimates divided by the phenotypic standard deviation were equal to − 4.023, − 1.702, − 2.516, 3.261, and − 2.197 for ADG, BF, LMD, AGE100, and TNB, respectively.

### Predictive abilities

The predictive abilities of the genomic models are in Table 5. For the four production traits (ADG, BF, LMD, and AGE100), the three models without inbreeding depression effects (MA, MAD and MAD*) showed similar predictive abilities. This was also true for the three models with inbreeding depression effects (MAI, MAID and MAID*). These results indicated that the inclusion of dominance effects in the model did not improve their predictive ability. However, for TNB, including dominance effects resulted in a 6.3% increase (from 0.167 to 0.177 for the MAD model and to 0.178 for the MAD* model) in predictive ability compared with the model with only additive effects (MA). In addition, for all the traits, the model with both additive effects and inbreeding depression effects (MAI) outperformed the model with only additive effects (MA), showing an ~ 1.7% increase in predictive ability.

**Table 5:** Accuracies of predicted total genetic values in the validation dataset for models MA, MAD, MAD*, MAI, MAID and MAID*

Evaluation model	ADG	BF	LMD	AGE100	TNB
MA	0.286	0.335	0.250	0.228	0.167
MAD	0.286	0.336	0.250	0.229	0.177
MAD*	0.287	0.336	0.249	0.23	0.178
MAI	0.292	0.338	0.252	0.233	0.172
MAID	0.292	0.337	0.252	0.234	0.180
MAID*	0.293	0.337	0.251	0.235	0.181

ADG: average daily gain; BF: backfat thickness; LMD: loin muscle depth; AGE100: days to 100 kg; TNB: total number of piglets born at first parity

Overall, the models with additive effects and inbreeding effects or dominance effects usually outperformed the model with only additive effects (MA). For the production traits, compared to model MA, the predictive ability was increased by ~ 1.5% with model MAI, ~ 1.5% with model MAID, and ~ 1.6% with model MAID*. For TNB, the predictive ability increased by ~ 6.0% with model MAD, ~ 6.6% with model MAD*, ~ 3.0% with model MAI, ~ 7.8% with model MAID, and ~ 8.4% with model MAID*. In terms of the regression coefficients of the corrected phenotypes on estimated genetic values (Table 6), the regression coefficient of model MA showed the greatest deviation from 1 compared to the other models. Most traits showed regression coefficients close to 1 for all models, except for BF and LMD, which had regression coefficients equal to 0.64 and 0.80, respectively. This result was unexpected, and we do not have a clear explanation for it.

**Table 6:** Regression coefficients of corrected phenotype on predicted total genetic values in the validation dataset for models MA, MAD, MAD*, MAI, MAID and MAID*

Evaluation model	ADG	BF	LMD	AGE100	TNB
MA	0.969	0.641	0.803	0.935	1.016
MAD	0.973	0.643	0.805	0.941	1.069
MAD*	0.974	0.643	0.802	0.945	1.073
MAI	0.985	0.644	0.808	0.954	1.035
MAID	0.986	0.646	0.809	0.955	1.073
MAID*	0.986	0.645	0.806	0.958	1.077

ADG: average daily gain; BF: backfat thickness; LMD: loin muscle depth; AGE100: days to 100 kg; TNB: total number of piglets born at first parity

In addition, we explored the accuracies of the genomic estimated breeding values (GEBV). As shown in Table 7, regardless of the model used, the accuracies of the GEBV were almost the same for all models and all traits. For the regression coefficients of corrected phenotypes on GEBV (Table 8), there was no clear trend of one model outperforming the others.

**Table 7:** Accuracies of predicted breeding values in the validation dataset for models MA, MAD, MAD*, MAI, MAID and MAID*

Evaluation model	ADG	BF	LMD	AGE100	TNB
MA	0.286	0.335	0.25	0.228	0.167
MAD	0.285	0.335	0.249	0.226	0.165
MAD*	0.286	0.336	0.25	0.227	0.169
MAI	0.286	0.335	0.248	0.225	0.168
MAID	0.286	0.335	0.248	0.224	0.166
MAID*	0.286	0.336	0.248	0.224	0.169

ADG: average daily gain; BF: backfat thickness; LMD: loin muscle depth; AGE100: days to 100 kg; TNB: total number of piglets born at first parity

**Table 8:** Regression coefficients of corrected phenotype on predicted breeding values in the validation dataset for models MA, MAD, MAD*, MAI, MAID and MAID*

Evaluation model	ADG	BF	LMD	AGE100	TNB
MA	0.969	0.641	0.803	0.935	1.016
MAD	0.978	0.650	0.814	0.959	1.108
MAD*	0.970	0.643	0.805	0.934	1.029
MAI	0.964	0.642	0.803	0.934	1.017
MAID	0.968	0.649	0.812	0.944	1.102
MAID*	0.964	0.642	0.804	0.933	1.029

ADG: average daily gain; BF: backfat thickness; LMD: loin muscle depth; AGE100: days to 100 kg; TNB: total number of piglets born at first parity

## Discussion

In this study, first we investigated different strategies to combine the genomic information from two SNP arrays. Subsequently, we examined the impact of the nonadditive effects on genomic predictive ability and further explored two models of fitting dominance effects (classical and genotypic) in the genomic prediction models. For the five traits studied, fitting inbreeding depression effects yielded the highest predictive abilities, and for one trait (TNB), the inclusion of dominance effects in the genomic model slightly increased the predictive ability.

### Imputation accuracy

The imputation accuracy in Scenario 1 was slightly higher than in Scenario 2. Previous studies have shown that imputation accuracy is affected by the MAF of the imputed SNPs and by the top relatedness between animals in the reference and imputed populations [31–35]. Thus, we investigated the distribution of the MAF of imputed SNPs and studied the highest relatedness of individuals between the imputed and reference populations. The proportion of SNPs with a low MAF was lower in Scenario 1 than in Scenario 2 (see Additional file 2: Figure S1), and the top genomic relatedness was slightly lower in Scenario 2 than in Scenario 1 (see Additional file 1: Table S6), which would probably lead to a higher imputation accuracy in Scenario 1.

### Estimated variance components

In this study, the estimated narrow-sense heritability confirmed that ADG, BF, LMD, and AGE100 were moderately heritable and that TNB was lowly heritable, in line with many other reports [13, 15, 36]. No significant difference in narrow-sense heritability was observed among the genomic models, which indicates that the additive genetic variance was accurately separated from the phenotypic variance in all genomic models, regardless of the nonadditive effects.

In this study, the proportions of dominance variation to the total genetic variance in production traits were relatively low (ranging from 1.9 to 10.5%) and generally lower than those found in other studies on production traits in pigs [8, 16]. The proportion of genotypic dominance variations relative to the total genetic variance of TNB was moderate (ranging from 18.2 to 20.3%) and was similar to that reported in a previous study [17]. Our finding that the proportion of genotypic dominance variations relative to the total genetic variance of TNB (20.3%) was higher than that for the production traits (~ 8.5%) in Yorkshire pigs was consistent with a previous study that reported that the proportion of classical dominance variation relative to the total genetic variance for another reproduction trait (calving interval) was ~ 34.3%, whereas that for production traits (milk, fat, and protein yields) was ~ 8.5% on average in Holstein cattle [14]. For all traits, there were no significant differences between the proportions of classical and genotypic dominance variation when standard errors were taken into account. One possible reason could be that the dominance variance was too small to distinguish between its two forms, and therefore this needs to be further investigated. Our data showed that although both classical dominance variance and genotypic dominance variance were small, the genotypic dominance variance was slightly larger than the classical dominance variance, as reported by Vitezica et al. [9]. Based on the conversion method described by Vitezica et al. [9], the estimated genotypic dominance variance can be easily converted into that obtained via the classical approach. As shown in Additional file 1: Table S7, after transformation, the estimated genetic variances from the genotypic dominance model (MAID) were close to those obtained from the classical dominance model (MAID^*^), which confirmed the equivalence of the estimates of dominant variation generated in this study. The standard error of the estimates of dominance variation was still relatively large, which indicates that the size of our dataset was not sufficient to accurately estimate dominance variation. Therefore, more data are needed to further investigate the dominance effects in the current population.

In this study, we used the pre-corrected phenotypes of the genotyped pigs as the response variables to estimate dominance variances. These genotyped pigs were not randomly sampled from the population, and most of them showed high EBV and were selected as parents for producing the next generation. Xiang et al. [18] reported that preselection and precorrection greatly reduced the variances of the dominance effects. In addition, putative errors in the imputed genotypes might increase the uncertainty of genomic evaluations [37]. It should be noted that in some other studies, the proportion of dominance variation to total genetic variance was found to be lower than in our study and even closer to 0 [38, 39]. Previous studies have shown that the proportion of dominance variation to total genetic variance is affected by various factors, i.e., the studied population, the target traits, types of information, and genomic models [8, 16]. Thus, more studies are needed to further investigate the effect of various factors on dominance variation.

### Estimates of inbreeding depression

As shown in Table 4, there were no large differences in the estimates of inbreeding depression parameters among the MAI, MAID and MAID* models when standard errors were taken into account, which is in line with previous studies [14, 18]. The estimates of inbreeding depression showed that inbreeding depression had detrimental effects on ADG, LMD, AGE100, and TNB, thus should be included in the model for genetic evaluation [30]. Inbreeding depression estimates for the same traits from previous studies [18, 19, 30, 36, 40] were similar to our results. For BF, inbreeding depression (negative value) did not show a detrimental effect in this study, in agreement with results on Pietrain pigs reported in [28]. For the BF trait in model MAI, we estimated a $\eta$ value of − 4.749, which means that an increase of 0.10 unit in the inbreeding coefficient led to a decrease of 0.479 mm in backfat thickness. Another study reported that inbreeding depression had no effect on backfat [41], and the authors attributed this to the change in dominance effect values across genes, suggesting that dominance effects at different loci might be either positive or negative [23]. Notably, the standard errors of the backfat estimates were large in our study, and the estimates of dominance effects of BF only slightly differed from 0. Therefore, larger datasets are needed to further investigate the inbreeding depression effects of BF.

The ratio of the estimated inbreeding depression effect divided by the phenotypic standard deviation for the trait is an indicator of the importance of the inbreeding depression effect [30]. In model MAI, for the ADG, LMD, AGE100, and TNB traits, the absolute values of this ratio were equal to 4.023, 2.516, 3.261, and 2.197, respectively. Note that the estimate of this effect refers to an individual with 100% inbreeding. For BF, the absolute value of the ratio was 1.702, which showed that inbreeding depression had little impact on BF. This phenomenon was consistent with the above findings.

Our study is the first to report the proportion of additive genetic effects that is contributed by inbreeding depression effects. According to the formula for calculating the additive variance, the proportion contributed by inbreeding depression is mainly affected by allelic frequencies, the magnitude of the estimated inbreeding depression parameter, and the number of SNPs used. As shown in Additional file 2: Figure S2, for a single locus, the value of ${2{p}_{j}{q}_{j}\left({q}_{j}-{p}_{j}\right)}^{2}$ is largest when the frequency of the reference allele is approximately 0.15. However, even if the frequency of the reference allele was 0.15 for all loci, the proportion of additive variance contributed by inbreeding depression would not change much since it needs to be divided by the number of SNPs used, $m$. This explains why the proportion of additive variance contributed by inbreeding depression was small for all traits in this study.

Overall, the inclusion of the inbreeding depression effect in the genomic model had no significant effect on the estimation of variance components for all traits, although all of the dominance variances were slightly reduced, as also reported by Aliloo et al. [14].

### Predictive abilities

The goodness-of-fit of the six genomic models showed that those with inbreeding depression effects (MAI, MAID, and MAID*) presented a better goodness-of-fit than the model with only additive effects (MA) for all traits, in line with Aliloo et al. [14]. This result suggests that inbreeding depression had an impact on the production traits and TNB, and thus this effect should be explicitly fitted in genomic evaluation models. This was confirmed by the results regarding predictive ability. Previous studies have reported that including dominance effects in a genomic model can improve its predictive abilities [8, 11, 15]. However, our study showed that including dominance effects in the genomic model only slightly improved predictive abilities for TNB. This might be related to the degree to which traits are affected by dominant genes. The observation that including inbreeding depression in the model improved the predictive ability whereas including dominance effects did not was also reported by Xiang et al. [18] and Aliloo et al. [14]. Our explanation for this finding is that dominance has two components that can be modeled separately [18]. The first is the directional dominance effect [18], which accumulates across loci and leads to an inbreeding depression effect that is modeled via a single covariate, with an accurately estimated effect. For the remaining residual dominance effects (which show a mean of zero), it is difficult to obtain accurate estimates using a dominance relationship matrix, especially when the sample size is not sufficient. Thus, even when dominance deviations were included in the genomic model, predictive abilities were not further improved. However, our study showed that although including dominance effects in the model did not improve its predictive ability for production traits, it did not decrease them either, which agrees with the results of a study on the total number of piglets born to Danish Yorkshire pigs [18].

## Conclusions

Our results revealed that the inclusion of an inbreeding depression effect in the genomic model increased its predictive ability for the four production traits (ADG, BF, LMD, and AGE100) and the reproduction trait (TNB) studied and that when the tested trait was strongly affected by dominance genes, the inclusion of the dominance effect in the model further improved its predictive ability. Even when the trait was little affected by dominance, the inclusion of the dominance effect in the model did not decrease its predictive ability.

## Supplementary Information

**Additional file 1: Table S1.** Estimates of variance components, standard error (SE) of parameters, − 2 log likelihood (− 2LogL), AIC (Akaike’s Information Criterion) from models MA, MAD, MAD*, MAI, MAID and MAID*. **Table S2.** Contributed additive genetic variance from the inbreeding depression effect. **Table S3.** P-value of likelihood ratio test based on model MA. **Table S4.** P-value of likelihood ratio test based on models MAI, MAD and MAD*. **Table S5.** P-value of inbreeding depression effect based on the Wald test. **Table S6.** Average genomic relationships between animals in the imputed and reference sets. **Table S7.** Converted classical variance components based on the genotypic variance component in models MAD and MAID.

**Additional file 2: Figure S1.** Distribution of the minor allele frequencies of the Illumina array-specific SNPs in Scenario 1 and the Affymetrix array-specific SNPs in Scenario 2. **Figure S2.** Effect of allele frequency on ${2{p}_{j}{q}_{j}\left({q}_{j}-{p}_{j}\right)}^{2}$.

## Data Availability

The genotypes and phenotypes used in the current study were generated from commercial farms and are not publicly available.
