# Supplementary material for: Impacts of additive, dominance, and inbreeding depression effects on genomic evaluation by combining two SNP chips in Canadian Yorkshire pigs bred in China
Source: Genet Sel Evol. 2022 Oct 22;54:69. doi: 10.1186/s12711-022-00760-4 (PMC9588241; doi:10.1186/s12711-022-00760-4)
Supplement: Supplementary file 1 — Additional file 1: Table S1. Estimates of variance components, standard error (SE) of parameters, − 2 log likelihood (− 2LogL), AIC (Akaike’s Information Criterion) from models MA, MAD, MAD*, MAI, MAID and MAID*. Table S2. Contributed additive genetic variance from the inbreeding depression effect. Table S3. P-value of likelihood ratio test based on model MA. Table S4. P-value of likelihood ratio test based on models MAI, MAD and MAD*. Table S5. P-value of inbreeding depression effect based on the Wald test. Table S6. Average genomic relationships between animals in the imputed and reference sets. Table S7. Converted classical variance components based on the genotypic variance component in models MAD and MAID. [file 12711_2022_760_MOESM1_ESM.docx]

**Table S1 Estimates of variance components, standard error(SE) of parameters, -2 log likelihood (-2LogL), AIC (Akaike’s Information Criterion) from models MA, MAD, MAD*, MAI, MAID and MAID*.**

| **Trait** | **Model** | $\boldsymbol{\sigma}_{\boldsymbol{a}}^{\boldsymbol{2}}\boldsymbol{(SE)}$ | $\boldsymbol{\sigma}_{\boldsymbol{i}}^{\boldsymbol{2}}$ | ${\boldsymbol{\sigma}_{\boldsymbol{d}}^{\boldsymbol{2}}\boldsymbol{(SE)\vert}\boldsymbol{\sigma}}_{\boldsymbol{d}^{\boldsymbol{*}}}^{\boldsymbol{2}}\boldsymbol{(SE)}$ | $\boldsymbol{\sigma}_{\boldsymbol{e}}^{\boldsymbol{2}}\boldsymbol{(SE)}$ | $\frac{\boldsymbol{\sigma}_{\boldsymbol{d}}^{\boldsymbol{2}}}{\boldsymbol{\sigma}_{\boldsymbol{g}}^{\boldsymbol{2}}}\boldsymbol{\vert}\frac{\boldsymbol{\sigma}_{\boldsymbol{d}^{\boldsymbol{*}}}^{\boldsymbol{2}}}{\boldsymbol{\sigma}_{\boldsymbol{g}}^{\boldsymbol{2}}}$ | **-2LogL** | $\mathbf{AIC}$ |
| --- | --- | --- | --- | --- | --- | --- | --- | --- |
| ADG | MA | 862.853(73.256) |  |  | 2475.376(50.657) |  | 58876.117 | 58880.117 |
|  | MAD | 851.607(73.131) |  | 34.185(37.849) | 2452.386(55.707) | 0.039 | 58875.724 | 58881.724 |
|  | MAD* | 861.478(71.959) |  | 27.741(28.036) | 2449.072(56.149) | 0.031 | 58875.418 | 58881.418 |
|  | MAI | 871.153(73.629) | 2.622 |  | 2462.499(50.462) |  | 58851.997 | 58855.997 |
|  | MAID | 865.837(73.363) | 2.674 | 15.518(36.052) | 2452.703(55.339) | 0.018 | 58852.798 | 58858.798 |
|  | MAID* | 870.262(72.269) |  | 12.267(26.418) | 2451.527(55.706) | 0.014 | 58852.725 | 58858.725 |
| BF | MA | 2.99(0.208) |  |  | 5.035(0.107) |  | 18659.364 | 18663.364 |
|  | MAD | 2.929(0.208) |  | 0.148(0.089) | 4.948(0.119) | 0.048 | 18659.439 | 18665.439 |
|  | MAD* | 2.974(0.206) |  | 0.112(0.066) | 4.943(0.12) | 0.036 | 18659.391 | 18665.391 |
|  | MAI | 2.998(0.208) | 0.001 |  | 5.03(0.107) |  | 18654.683 | 18658.683 |
|  | MAID | 2.939(0.208) | 0.001 | 0.137(0.089) | 4.951(0.119) | 0.045 | 18654.145 | 18660.145 |
|  | MAID* | 2.981(0.206) |  | 0.106(0.065) | 4.946(0.12) | 0.034 | 18653.532 | 18659.532 |
| LMD | MA | 5.386(0.468) |  |  | 16.252(0.333) |  | 25783.73 | 25787.73 |
|  | MAD | 5.264(0.465) |  | 0.267(0.253) | 16.098(0.366) | 0.048 | 25785.292 | 25791.292 |
|  | MAD* | 5.358(0.458) |  | 0.198(0.186) | 16.089(0.369) | 0.036 | 25786.107 | 25792.107 |
|  | MAI | 5.344(0.466) | 0.007 |  | 16.244(0.332) |  | 25774.316 | 25778.316 |
|  | MAID | 5.242(0.463) | 0.007 | 0.232(0.251) | 16.113(0.366) | 0.042 | 25776.761 | 25782.761 |
|  | MAID* | 5.323(0.456) |  | 0.177(0.185) | 16.102(0.369) | 0.032 | 25777.602 | 25783.602 |
| AGE100 | MA | 14.344(1.32) |  |  | 51.361(1.035) |  | 33324.781 | 33328.781 |
|  | MAD | 13.786(1.33) |  | 1.617(0.852) | 50.28(1.146) | 0.105 | 33321.01 | 33327.01 |
|  | MAD* | 14.231(1.3) |  | 1.363(0.649) | 50.09(1.16) | 0.087 | 33319.926 | 33325.926 |
|  | MAI | 14.361(1.321) | 0.034 |  | 51.227(1.033) |  | 33309.238 | 33313.238 |
|  | MAID | 13.916(1.33) | 0.039 | 1.305(0.829) | 50.357(1.143) | 0.086 | 33307.073 | 33313.073 |
|  | MAID* | 14.272(1.301) |  | 1.106(0.629) | 50.198(1.156) | 0.072 | 33306.301 | 33312.301 |
| TNB | MA | 1.119(0.181) |  |  | 9.948(0.227) |  | 16342.066 | 16346.066 |
|  | MAD | 1.031(0.19) |  | 0.262(0.197) | 9.772(0.258) | 0.203 | 16340.597 | 16346.597 |
|  | MAD* | 1.114(0.18) |  | 0.157(0.145) | 9.799(0.261) | 0.123 | 16341.189 | 16347.189 |
|  | MAI | 1.131(0.182) |  |  | 9.932(0.227) |  | 16336.812 | 16340.812 |
|  | MAID | 1.05(0.191) | 0.003 | 0.233(0.195) | 9.777(0.257) | 0.182 | 16335.884 | 16341.884 |
|  | MAID* | 1.124(0.181) | 0.003 | 0.137(0.144) | 9.803(0.26) | 0.108 | 16336.349 | 16342.349 |

$\boldsymbol{\sigma}_{\boldsymbol{a}}^{\boldsymbol{2}}$: additive genetic variance; $\boldsymbol{\sigma}_{\boldsymbol{i}}^{\boldsymbol{2}}$: additive genetic variance contributed by inbreeding depression effects; $\boldsymbol{\sigma}_{\boldsymbol{d}}^{\boldsymbol{2}}$: genotypic dominance variance; $\boldsymbol{\sigma}_{\boldsymbol{d}^{\boldsymbol{*}}}^{\boldsymbol{2}}$: classical dominance variance; $\boldsymbol{\sigma}_{\boldsymbol{e}}^{\boldsymbol{2}}$:residual variance; $\boldsymbol{\sigma}_{\boldsymbol{g}}^{\boldsymbol{2}}$: total genetic variance;

MA: additive model; MAI: additive plus inbreeding depression model; MAD: additive plus genotypic dominance model; MAD*: additive plus classical dominance model; MAID: additive plus inbreeding depression plus genotypic dominance model; MAID*: additive plus inbreeding depression plus classical dominance model; ADG: average daily gain (g); BF: backfat thickness (mm); LMD: loin muscle depth (mm); AGE100: days to 100kg; TNB: total number of piglets born at first parity

**Table S2** **Contributed additive genetic variance from the inbreeding depression effect.**

| **Trait** | **Model** | $\boldsymbol{\sigma}_{\boldsymbol{a}}^{\mathbf{2}}$ | $\boldsymbol{\sigma}_{i}^{\mathbf{2}}$ | 100*$\frac{\boldsymbol{\sigma}_{\boldsymbol{i}}^{\mathbf{2}}}{\boldsymbol{\sigma}_{\boldsymbol{a}}^{\mathbf{2}}+\boldsymbol{\sigma}_{\boldsymbol{i}}^{\mathbf{2}}}$ |
| --- | --- | --- | --- | --- |
| ADG | MAI | 871.153 | 2.622 | 0.300 |
|  | MAID | 865.837 | 2.674 | 0.308 |
| BF | MAI | 2.998 | 0.001 | 0.037 |
|  | MAID | 2.939 | 0.001 | 0.031 |
| LMD | MAI | 5.344 | 0.007 | 0.125 |
|  | MAID | 5.242 | 0.007 | 0.130 |
| AGE100 | MAI | 14.361 | 0.034 | 0.236 |
|  | MAID | 13.916 | 0.039 | 0.282 |
| TNB | MAI | 1.131 | 0.003 | 0.232 |
|  | MAID | 1.05 | 0.003 | 0.273 |

$\boldsymbol{\sigma}_{\boldsymbol{a}}^{\boldsymbol{2}}$: additive genetic variance; $\boldsymbol{\sigma}_{\boldsymbol{i}}^{\boldsymbol{2}}$: contributed additive genetic variance from inbreeding depression effect;

MAI: additive plus inbreeding depression model; MAID: additive plus inbreeding depression plus genotypic dominance model; ADG: average daily gain (g); BF: backfat thickness (mm); LMD: loin muscle depth (mm); AGE100: days to 100kg; TNB: total number of piglets born at first parity;

**Table S3** **P-value of likelihood ratio test based on model MA.**

| **Trait** | **MAI** | **MAD** | **MAD*** |
| --- | --- | --- | --- |
| ADG | 4.53E-07 | 0.265 | 0.202 |
| BF | 0.015 | 0.500 | 0.500 |
| LMD | 0.001 | 0.500 | 0.500 |
| AGE100 | 4.03E-05 | 0.026 | 0.014 |
| TNB | 0.011 | 0.113 | 0.175 |

ADG: average daily gain (g); BF: backfat thickness (mm); LMD: loin muscle depth (mm); AGE100: days to 100kg; TNB: total number of piglets born at first parity;

**Table S4** **P-value of likelihood ratio test based on models MAI, MAD and MAD*.**

| **Trait** | **MAI vs MAID** | **MAI vs MAID*** |
| --- | --- | --- |
| ADG | 0.500 | 0.500 |
| BF | 0.232 | 0.142 |
| LMD | 0.500 | 0.500 |
| AGE100 | 0.071 | 0.043 |
| TNB | 0.168 | 0.248 |

ADG: average daily gain (g); BF: backfat thickness (mm); LMD: loin muscle depth (mm); AGE100: days to 100kg; TNB: total number of piglets born at first parity;

**Table S5** **P-value of inbreeding depression effect based on the Wald test.**

| **Trait** | **MAI vs MA** | **MAID vs MAD** | **MAD* vs MAID*** |
| --- | --- | --- | --- |
| ADG | 8.77E-07 | 1.68E-06 | 1.80E-06 |
| BF | 0.030 | 0.020 | 0.015 |
| LMD | 0.002 | 0.004 | 0.004 |
| AGE100 | 7.99E-05 | 1.92E-04 | 2.26E-04 |
| TNB | 0.022 | 0.030 | 0.028 |

ADG: average daily gain (g); BF: backfat thickness (mm); LMD: loin muscle depth (mm); AGE100: days to 100kg; TNB: total number of piglets born at first parity;

**Table S6** **Average genomic relationships between animals in the imputed and reference sets.**

| **Scenario** |  | **Genomic Relationship** | | |
| --- | --- | --- | --- | --- |
|  |  | **Top1** | **Top5** | **Top10** |
| Scenario1 |  | 0.274 | 0.181 | 0.153 |
| Scenario2 |  | 0.237 | 0.178 | 0.152 |

**Table S7 Co****nverted classical variance components based on the genotypic variance component in models MAD and MAID.**

| **Model** | **Trait** | $\boldsymbol{\sigma}_{\boldsymbol{a}}^{\boldsymbol{2}}$ **_(converted)_** | $\boldsymbol{\sigma}_{\boldsymbol{d}}^{\boldsymbol{2}}$ **_(converted)_** |
| --- | --- | --- | --- |
| MAD | ADG | 861.987 | 23.805 |
|  | BF | 2.974 | 0.103 |
|  | LMD | 5.345 | 0.186 |
|  | AGE100 | 14.277 | 1.126 |
|  | TNB | 1.107 | 0.243 |
| MAID | ADG | 870.549 | 10.806 |
|  | BF | 2.981 | 0.095 |
|  | LMD | 5.312 | 0.162 |
|  | AGE100 | 14.312 | 0.909 |
|  | TNB | 1.115 | 0.229 |

ADG: average daily gain; BF: backfat thickness; LMD: loin muscle depth; AGE100: days to 100kg; TNB: total number of piglets born at first parity;

$\boldsymbol{\sigma}_{\boldsymbol{a}}^{\mathbf{2}}$(converted): Converted classical additive genetic variance based on genotypic model;

$\boldsymbol{\sigma}_{\boldsymbol{d}}^{\mathbf{2}}$(converted): Converted classical dominance genetic variance based on genotypic model;
