# Supplementary material for: Impacts of additive, dominance, and inbreeding depression effects on genomic evaluation by combining two SNP chips in Canadian Yorkshire pigs bred in China
Source: Genet Sel Evol. 2022 Oct 22;54:69. doi: 10.1186/s12711-022-00760-4 (PMC9588241; doi:10.1186/s12711-022-00760-4)

**Figure S1** **Distribution of the minor allele frequencies of the Illumina array-specific SNPs in Scenario 1 and the Affymetrix array-specific SNPs in Scenario 2.**


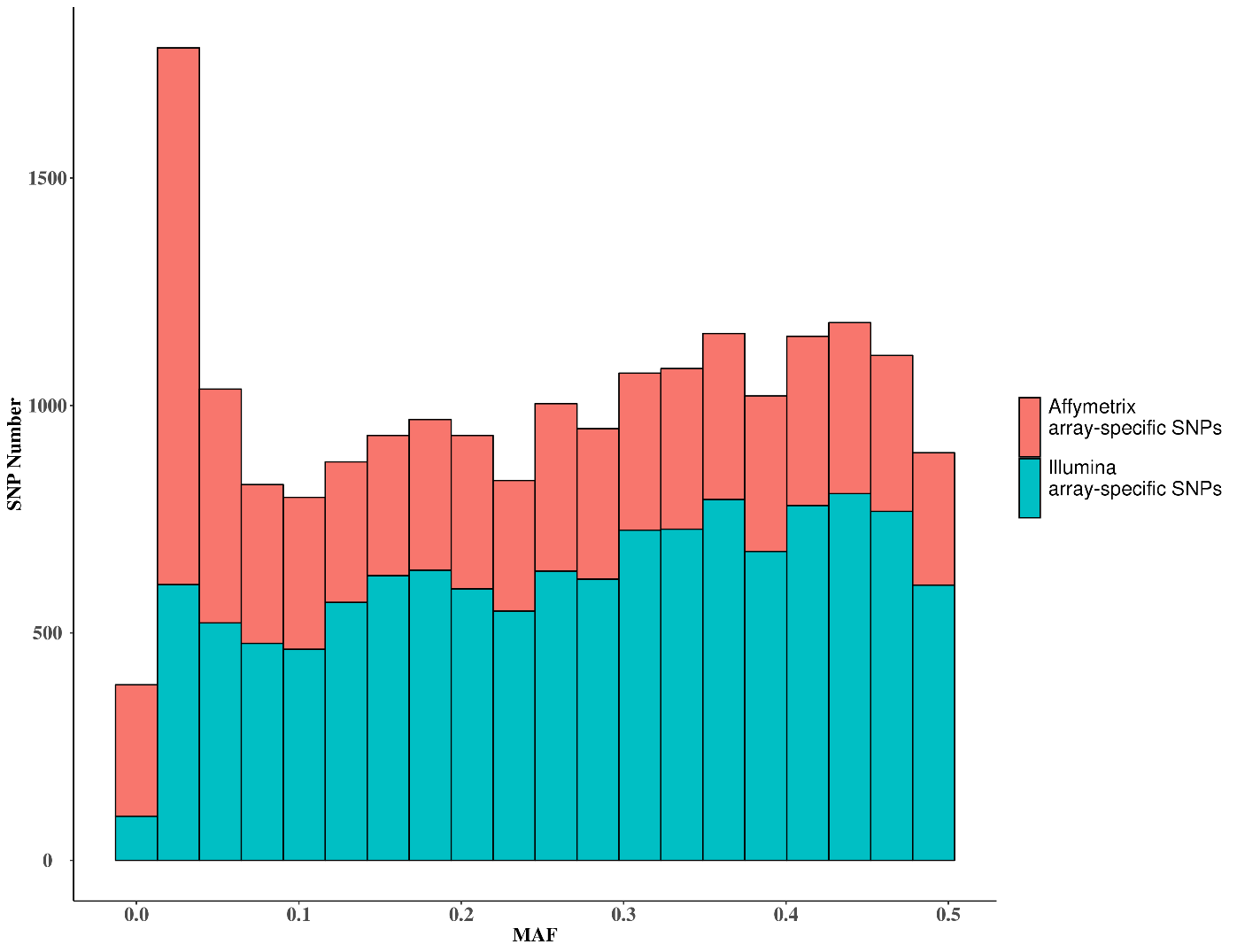


**Figure S2** **Effect of allele frequency on** ${\mathbf{2}\boldsymbol{p}_{\boldsymbol{j}}\boldsymbol{q}_{\boldsymbol{j}}\left( \boldsymbol{q}_{\boldsymbol{j}}\mathbf{-}\boldsymbol{p}_{\boldsymbol{j}} \right)}^{\mathbf{2}}$**.**


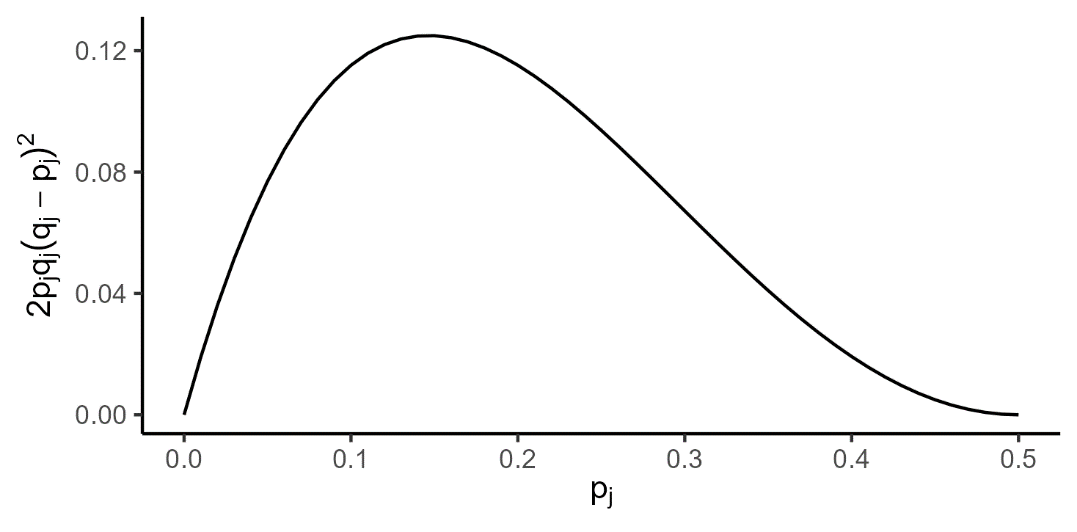

Supplement: Supplementary file 2 — Additional file 2: Figure S1. Distribution of the minor allele frequencies of the Illumina array-specific SNPs in Scenario 1 and the Affymetrix array-specific SNPs in Scenario 2. Figure S2. Effect of allele frequency on ${2{p}_{j}{q}_{j}\left({q}_{j}-{p}_{j}\right)}^{2}$2pjqjqj-pj2. [file 12711_2022_760_MOESM2_ESM.docx]
